# Development of a prostate cancer survivorship care toolkit for Black men with clinical and contextual tailoring

**DOI:** 10.1007/s00520-026-11026-w

**Published:** 2026-07-23

**Authors:** Kimlin Ashing, Floyd Willis, Christopher Williams, James Morrison, John McCall, Gaole Song, Fornati Bedell, Cassandra Moore, Ewan Cobran, Che Ngufor, Timethia Bonner, Getachew Dagne, Arnold Merriweather, JoAnne Oliver, Ernie Kaninjing, Rotimi Oladapo, Roxana Dronca, Folakemi Odedina

**Affiliations:** 1 City of Hope Comprehensive Cancer Center, Duarte, CA USA; 2 Africa Carribean Consortium, Jacksonville, FL USA; 3 Mayo Clinic Comprehensive Cancer Center, Jacksonville, FL USA; 4 iCCaRE for Black Men Consortium, Mayo Clinic, Jacksonville, FL USA; 5 Bethel Missionary Baptist Church, Pasadena, CA USA; 6 Mayo Clinic Comprehensive Cancer Center, Phoenix, AZ USA; 7 Mayo Clinic Comprehensive Cancer Center, Rochester, MN USA; 8 University of South Florida, Tampa, FL USA; 9 Legion Post 197, Jacksonville, FL USA; 10 University of Alabama, Tuscaloosa, AL USA; 11 Georgia College, Milledgeville, GA USA; 12 Prostate Cancer Transatlantic Consortium (CaPTC), Mayo Clinic, Jacksonville, FL USA; 13 Covenant University of Nigeria, Ota, Ogun State Nigeria

**Keywords:** Black men, Prostate cancer, Survivorship care plan, Health-related quality of life, Cancer disparities

## Abstract

**Purpose:**

Black men experience the highest prostate cancer (CaP) burden among all racial and ethnic groups, underscoring the urgent need for research to guide the development and implementation of culturally tailored strategies aimed at reducing disparities and improving health outcomes. Survivorship care resources are recommended to educate patients and provide them with better information about their cancer diagnosis, treatment, and follow-up care, thereby empowering them to be active participants in their own survivorship care for improved quality of life and well-being. Due to the lack of culturally relevant survivorship care, we aimed to develop a survivorship care toolkit (SCT) that includes a survivorship care roadmap template tailored to Black men to address the unique survivorship care needs of Black prostate cancer survivors (CaPS). Our approach is grounded in the science of survivorship and rooted in the science of community engagement, which requires a genuine, bidirectional relationship between scientists and survivor-advocates.

**Methods:**

Following literature and guideline review of the American Society of Clinical Oncology (ASCO) survivorship care template, we employed a three-pronged method to develop the toolkit that included a literature review, a consensus-building approach with diverse stakeholders, and in-depth interviews with Black prostate cancer survivors (CaPS).

**Results:**

Following community-engaged research, we included 22 diverse Black CaPS to review and tailor the SCT. Results supported the enhancement of the ASCO template by incorporating and expanding domains including family history, care team contacts, follow-up and surveillance, symptom management, quality of life, survivor-specific concerns, major comorbidities, health advisories, and culturally relevant resources.

**Conclusions:**

Black CaPS expressed satisfaction with the tailored SCT as a tool and a credible, clinically, and community-responsive resource to improve follow-up and survivorship care, patient documentation, activation, and communication about their survivorship concerns and needs.

## Background

Prostate cancer (CaP) is the second most common cancer among men, with approximately 1 in 8 men in the USA receiving a formal diagnosis annually [1–3]. Prostate cancer survivorship is defined as any person from the point of a cancer diagnosis through the balance of life. Advances in treatment have led to significant increases in survival rates, with over 90% of men diagnosed with prostate cancer living at least 5 years or more [4–6]. However, surviving cancer and its treatment often weighs a hefty survivorship burden, with profound survivorship differences borne by vulnerable cancer survivors. Black men experience a disproportionately higher incidence and mortality rate from CaP compared to White men, leading to a significantly greater burden of the disease. Further, Black men are 1.76 times more likely to be diagnosed with CaP and 2.2 times more likely to die from it compared to White men [1]. Disparities that black men experience include diagnosis at a more advanced stage, greater morbidity and mortality, and poorer survivorship outcomes than other races [7]. The priority population burdens are well documented for higher incidence, advanced stages, and death rate [8–10], and these CaP survivors (CaPS) also suffer higher treatment-related side-effects leading to worse survivorship outcomes, such as lower quality of life and heavier distress burdens, than other populations [11–13]. During and after treatments, these survivors often experience elevated physical, functional, sexual, and emotional symptom burden and declines that contribute to a high unmet survivorship care need [14–17]. Due to growing patient complexity and fragmentation of the US healthcare system, cancer survivors experience decreased access and unmet needs, with the greatest gaps incurred by survivors from the vulnerable populations [18–22]. A survivorship care plan is a personalized document for cancer survivors, created upon finishing treatment, that outlines their diagnosis, treatment history, and a roadmap for future health [2, 3]. Research focused more on improving cancer prevention and treatment; limited studies addressed the issue that there are no suitable survivorship care resources tailored for Black CaPS, which we have discussed in our previous studies [10–18]. Yet, survivorship is understudied, and research aimed at tackling survivorship improvements focused on Black men is lacking. To address the undue burden borne by Black men, a transdisciplinary, multisectoral partnership of clinicians, researchers, and community advocates formed the inclusive Cancer Care Research Equity (iCCaRE) Consortium for Black Men. The iCCaRE Consortium prioritizes the relationships with community leaders and advocates to bridge the gap between the medical system and the community. The consortium focuses on identifying gaps in care, reducing disparities, and advancing health equity for Black men with prostate cancer. An essential pillar of the consortium is HRQOL and survivorship—a critical patient-centered aspect not adequately studied and often overlooked, especially with Black CaP Survivors (CaPS).

In response to these notable gaps and in collaboration with the iCCaRE Consortium, the objective of this study is to address survivorship care improvements by developing a survivorship care toolkit (SCT) tailored to Black prostate cancer survivors.. This study is rooted in the science of survivorship (SOS) [23] and the contextual model of survivorship [24]. SOS and the contextual model of survivorship were used as theoretical frameworks in this study since they examine complex factors influencing a cancer survivors’ quality of life, taking into account not only individual characteristics but also broader social, cultural, and healthcare system contexts that significantly impact their experience post-treatment. Essentially, the SOS and the contextual model view survivorship as a dynamic process shaped by multiple interacting variables across different levels, from personal to systemic, which align with the goals of this study and inform the study design [25–27]. Importantly, the science of community engagement provided the guidelines for the approach to engage and rely on the experience of multisectoral stakeholders, e.g., clinicians, advocates, and survivors’ lived experiences. This study aims to improve Black prostate cancer survivors’ cancer literacy, follow-up care, self-care management, and QOL (physical, functional, emotional, social, and spiritual) well-being outcomes.

## Methods

### Methodology for CaP-SCT development

This study developed a survivorship care toolkit (SCT) tailored to ethnically diverse Black men, using a three-pronged method that included a literature review, a consensus-building approach to conduct in-depth interviews with diverse stakeholders (including clinicians, community health workers, advocates), and one-on-one, in-depth interviews with Black prostate cancer survivors. Based on the literature review, the American Society of Clinical Oncology (ASCO) Prostate Cancer Care Plan template [3] was selected as the foundation for our CaP-SCT. The ASCO SCP template is recognized as the industry standard.

The study team identified domains and content to clinically and culturally tailor the original ASCO Survivorship Care Plan (ASCO-SCP) to better address the complete individual, whole-person, and unique needs of Black survivors. We held a series of consensus-building discussions with clinicians and members from the Inclusive Cancer Care Research Equity for Black Men Consortium (iCCaRE) to guide and refine the template, ensuring clinical and cultural relevance, as well as conciseness, literacy, and functionality. These multisectoral stakeholders included three prostate cancer physicians, a general practice physician, a psych-oncologist, a doctoral-level oncology nurse, behavioral scientists, and survivor-advocates. Consensus-building meetings were conducted with iCCaRE’s survivor partners and co-investigators. The consensus approach aimed to enhance the SCT’s responsiveness to the unique experiences of Black CaPS. These multisectoral stakeholders emphasized the SOS and embraced the self-care model approach.

Therefore, with input from the iCCaRE Consortium, the tailored SCT template addresses the informational, clinical, and quality-of-life needs of Black CaPS. Employing this consensus approach, the team revised the ASCO-SCP by incorporating content that supports comprehensive post-treatment care tailored to Black men who suffer the greatest CaP survivorship burden and survivorship care disparities. Feedback from the stakeholders and CaP survivors supported the final version of the CaP-SCT template. After six rounds of input and iterations, this collaborative consensus method resulted in a draft CaP-SCT, which was then piloted and evaluated by CaPS (*N* = 22).

### Methodology for CaPS review and pilot of the prototypical SCT-CaPS

The study was approved by the Institutional Review Boards of City of Hope and the Institutional Review Boards of the Mayo Clinic. This study was performed in line with the principles of the Declaration of Helsinki, CIOMS, Belmont Report, and US Common Rule. Informed consent was obtained from all individual participants included in the study. Every participant provided their signed consent form (handwritten or digital, based on participants’ preference).

### Participant enrollment

The participant enrollment was from January 1, 2023, to December 30, 2024. The inclusion criteria for participation were as follows: (1) self-identifying as Black, (2) diagnosed with non-metastatic prostate cancer (stages 1–3) between January 1, 2019, and December 30, 2023, (3) ability to speak and understand English, (4) ownership of a standard smartphone with internet access and the ability to download applications, and (5) being at least 21 years of age.

Participants were recruited through community partners and hospital registries. The enrollment process included a detailed review of consent forms, during which participants’ questions were addressed, and concerns were clarified. Every participant provided their signed consent form (handwritten or digital, based on participants’ preference).

### Data collection

Participants received the CaP-SCT template and consent form digitally via email for review and were encouraged to reach out to the team with any questions. For participants without email access who wished to contribute, the documents were printed and mailed. Once the signed consent forms were received either digitally or via mail, participants were asked to choose between a Zoom call and an in-person interview based on their preference. While many participants were open to Zoom sessions, some faced challenges navigating the platform. In such cases, the project coordinator assisted participants in installing and using the Zoom software. Data collection included interviewer notes and participant-approved audio recording (including the Zoom recording) to ensure accuracy and thoroughness.

### Semi-structured motivational interview process for CaP-SCT

Motivational interviewing (MI) was utilized for the approach of the individual CaP-SCT education and utility session with the CaPS. Motivational interviewing emphasizes education and mindfulness of experiences and capacity building [28]. MI is a person-centered coaching approach designed to help a person with knowledge, skills, and practices to achieve a goal; it is collaborative, creating a safe space for a goal-oriented method that is solution-focused [29–31]. This approach was particularly suited to the use of a semi-structured interview protocol, allowing for the in-depth exploration of Black men’s experiences with CaP. Interviews were transcribed verbatim, with coding and thematic analysis connected to the following transcription. To enhance the depth of analysis, several active listening sessions of the interview recordings were conducted to capture nuances such as pauses and changes in tone during the interviews. Additionally, the interviewer recorded field notes after each interview, which were also used to inform the coding process.

The study aimed to develop an SCT template that addresses holistic care, considering cultural, clinical, and socioecological contexts. To achieve this, additional fields and information were identified and incorporated into the ASCO SCP template to enhance responsiveness to Black men. Based on the consensus process, the study team, including our survivor-advocate members and clinicians, agreed to retain all elements of the ASCO template with enhancements responsive to the cultural, clinical, and socioecological contexts of Black men. Similarities included the following: (1) contact information, (2) healthcare providers, (3) treatment summary, with types of treatment, e.g., chemotherapy, radiation therapy, hormonal therapy, and side effects, (4) cancer type, (5) stage and location, (6) Gleason score, (7) PSA at diagnosis, (8) genetic testing, (9) follow-up care and surveillance, and (10) coordinating provider.

Overall, our CaP-SCT emphasizes a patient activation approach [18–27]. Therefore, this study included CaPS education, communication with the care team, and self-management content. Specific enhancements included the following: (1)The general information section was updated to include patient ID and contact information for their family/caretaker, and the healthcare provider section includes a more comprehensive list of healthcare providers. (2) Treatment summary section, such as age at diagnosis, was included. (3) The types of therapy section now includes a list of side effects for each type of treatment and an echocardiogram for chemotherapy, a start/end date, region treated, and radiation dose, and lastly, for hormonal therapy; we included a reason for stopping/nonadherence to therapy. (4) Surveillance and follow-up include a more comprehensive list of medical exams and recommended tests. This was emphasized as a critical area to educate CaPS and document the needed surveillance for prostate cancer, as these men have the possibility of recurrence—a scenario that can occur even years after successful treatment and remission. Recognizing this, the consortium ensured that this consideration was thoughtfully integrated into the SCT. (5) Major comorbidities were added including common illnesses and medications. Also, a section on integrated primary care, cancer screenings, and coordinated cancer care. (6) Quality of life section now allows survivors to expand on areas of concern, which includes a rating scale with survivor concerns (e.g., anxiety, depression), an option to say if the care team is notified, and area for survivor notes. (7) We added a section on health advisories with daily healthy living recommendations, including nutrition, physical activity, and vaccinations. Self-management in healthcare is the ability to manage one’s health, including chronic conditions, by taking charge of one’s medical needs, daily responsibilities, and emotions. The goal of self-management is to improve quality of life and physical and emotional health and minimize disease-related impairments. Our SCT presented culturally appropriate ways to develop self-management skills including the following: setting realistic goals for care-team communication and healthy life style practices; practicing stress management techniques like breathing, exercise, or healthful eating; honoring emotions and prioritizing emotional well-being; being gentle with oneself and acknowledging to that the challenges that comes with cancer survivorship are real and fairly common; normalizing feeling while taking action; embracing strengths including support systems and persons and spirituality; and nurturing relationships with others. (8) The final section presented national cancer-related resources and specifically Black men’s health-focused resources. It is paramount to have these enhanced components to attend to the clinical, socioecological, and cultural lives of survivors to attend to the whole person care. A table outlining the components of the ASCO template and the added clinically and socioecologically responsive components in the iCCaRE SCT template is presented in Table 1.

**Table 1:** ASCO vs iCCaRE

SCT domains	Improvements
General information	
Contact information	Patient ID Support contact (name, number and relationship)
Healthcare providers	Added categories for: Mental health/social worker Nurse practitioner Primary care Urologist Other specialist
Treatment summary	
Cancer type, stage, and location	Age at diagnoses
PSA at diagnosis	
Clinical trials	Importance of Black inclusion clinical studies
Types of therapy	
List of surgeries and dates	Side effects
Chemotherapy	Type start/end date Dose Side effect list
Radiation therapy	Type start/end date Region treated Radiation dose
Hormonal therapy	Side effects Response
Follow-up care and surveillance	
Coordinated primary care	Physical exam schedule Hospitalization
Coordinated cancer care	Cancer screenings and other cancer diagnosis (date, stage, treatment, status)
Other recommended tests	Suggested follow-up tests listed, e.g., echocardiogram
Family history	1st-degree relatives with cancer
Major comorbidities	Included co-occurring illnesses and meds
Quality of life	
Treatment symptoms	Allows patients to expand on areas of concern Option to indicate if care team is notified
Rating scale	Allows patients to rate their concerns
Referral contact	Specialty care referral contact info
Survivor notes	Provides space of any added info
Health advisories	
Survivorship plan	Healthy lifestyle Healthcare, e.g., vaccinations, screenings
Resource information	List of cancer and Black health resources

## Results

### SCT evaluators participant demographics

We focused on the qualitative method to guide the evaluation of the SCT template. Three interviews were in-person, and 19 were conducted via Zoom. The age ranged from 48 to 80 among the 22 CaPS enrolled in the interview; 8 (42.1%) of them were diagnosed at stage 3, and 7 (31.8%) were at stage 2. The majority of the CaPS (68.2%) received surgery, and most of them (90.9%) reported having side effects from their CaP treatment. Additionally, 4 of them (18.2%) received genetic counseling and testing or genomic testing. From the aspect of CaP care access and utilization, most patients (94.7%) received timely healthcare services for their initial treatment. Almost 4 in 5 (78.9%) saw a doctor for follow-up CaP care and talked with doctors about their health in the past year and were seen in medical centers with specialized care. Cancer/Oncology Centers, including Comprehensive Cancer Centers, are the most common places. Still, 1 in 5 reported inadequate follow-up care. Table 2 shows the demographic information.

**Table 2:** Demographic information of participants

Characteristics	*N* (%)
Ethnicity	
*African American*	20 (90.9)
*Afro Caribbean*	1 (4.5)
*Other*	1 (4.5)
The stage of diagnosis	
*Stage 1*	3 (13.6)
*Stage 2*	7 (31.8)
*Stage 3*	8 (36.4)
*Stage 4*	4 (18.2)
CaP treatment type	
*Radiology therapy*	2 (9.1)
*Surgery*	15 (68.2)
*Hormonal surgery*	5 (22.7)
Had ever experienced any side effects from prostate cancer treatments	
*Yes*	20 (90.9)
*No*	2 (9.1)
Diagnosis of other medical conditions	
*Yes*	12 (54.5)
*No*	10 (45.5)
Had genetic or genomic testing	
*Yes*	4 (18.2)
*No*	18 (81.8)
During the past 12 months, have you seen a doctor for follow-up cancer care?	
*Yes*	18 (81.8)
*No*	4 (18.2)
During the past 12 months, have you seen or talked to any healthcare providers about your own health?	
*Yes*	18 (81.8)
*No*	4 (18.2)
Have you delayed getting care in the past 12 months?	
*Yes*	3 (13.6)
*No*	19 (86.4)
When did you last see any doctor for an oncology re-evaluation or cancer-related follow-up care?	
*Less than 3 months*	6 (27.3)
*More than 3 months but less than 6 months*	7 (31.8)
*More than 6 months but less than 1 year*	4 (18.2)
*More than 1 year*	5 (22.7)
What kind of place do you usually go to for cancer follow-up care?	
*County hospital*	0
*Community cancer/oncology practices*	1 (4.5)
*Private doctor’s office*	7 (31.8)
*Medical center with specialized cancer/oncology center including comprehensive cancer centers*	14 (63.6)

### Survivor CaP-SCT template feedback

Table 3 details the evaluation and feedback provided by the survivors. When asked about the various categories, there was a favorable acceptance of the intervention. Based on the survivors’ feedback, further enhancements for clarity and utility were made to the family history, care team contacts, follow-up and surveillance, quality-of-life symptoms, survivor-specific concerns, major comorbidities, and health advisories.

**Table 3:** Feedback from survivors

Section	CaP input and quote
General health information	“Not too bad to understand”
Family history	“And that was actually a concern of mine. Because I don’t, I don’t really know my family history. So, there were questions they were asking me that I couldn’t answer. Because I don’t know.”
Medical terminology/definitions	“Everything’s pretty self-explanatory”
Treatment	“I think that was clear’ and help me to keep track of all my complex treatment- surgery, radiation and hormonal.”
Follow-up and surveillance	“Since I do not have a medical background, going through this list about what to do and what should not do. So, I think that’s excellent.”
Care team	“Oh, I have all that support; good to know so others are aware they need a large team and make sure they keep all the contacts of everyone who treated them for the cancer and other things in one place (doc)”
Major chronic illnesses	“I think those things are valid, sure” We are aware that our community face more illnesses. The cancer drs do always ask so this help us the remind them (Drs) about all our ailments
Social determinants of health	“Well, a lot of those boxes were good, fairly good shape, you know. I took care of myself. Try to, anyway. Yeah, II mean, those are things. Some of those guys, logistically, could have issues, you know, getting tuned back for hospital finance would be a major hurdle for a lot of guys.”
Quality of life	“Yeah, so I’m good with it. It’s it’s excellent information, and it’s good to have and probably good to review for the person who has it to keep in mind for their immediate family…”
Health advisories	“Very good, Yeah, cuz I didn’t realize that I needed to change my diet.”
Resources	“Yes. Excellent” Good to have resources that focus on our community. They will understand us…the culture
Other	“I mean, if we have something like that (survivorship care plan)…. every time I pulled it up this was facing me in saying It doesn’t have to be a death sentence”

Attending and incorporating the feedback from the CaP-SCT template pilot implementation was essential for understanding how men wanted to be supported in their survivorship journey. One participant noted that the daily challenges of living with prostate cancer can take a toll on their mental well-being. One survivor suggested that researchers consider including “inspirational quotes” at the beginning of each section to help men navigate the mental struggles of everyday life. “I mean, if we have something like that (survivorship care plan)…. every time I pulled it upon my phone, this was facing me and saying it doesn’t have to be a death sentence.” This highlights that informational resources can also be a source of hope.

CaPS recommended adding it to the social support section of the health advisories “I thought that unless you’ve been through it’s hard to relate. You can read all the general stuff and, in the magazines, or on the internet, but I thought that could come in handy --to have like a support group like that --to someone…I wonder if that would be helpful.” One survivor (participant # 3) had a suggestion to include *journaling* in the Health Advisories section to reduce stress “You know, one of the things I did… I kept like a journal of all the things that happened each day and I gave it to my doctor, so they could keep it in the file. Because again, I may be hard to remember all your going thru and keep track.”

One survivor commented on expanding the major comorbidities section because some men might be reluctant to discuss their struggles: “Well some of themmay have psychological diagnosis… a lot of people are reluctant to answer anything about psychiatric, mental health … they’ll get real protective. They’ll say they’re fine when they’re actually suffering. Maybe say emotional health.” He also adds “And under substance abuse. Might you put recreational drug use. Substance abuse might automatically put a negative spin on it.”

A key factor discussed extensively was whether survivors could easily comprehend the document. Could they interpret and understand the language used? To address this, the consortium and PES CoRE carefully reviewed the content to ensure it was written at an appropriate reading level for all intended readers. We also included definitions and explanations for acronyms to provide clarity for those who might require additional support.

While some CaPS found the document to be lengthy, they acknowledged the importance of including comprehensive questions to support a holistic approach to care. One participant noted, “I think it’s good; I do not think it’s above the average person. Anybody with a high school education should be able to read this document.” This consideration also applied to understanding the layout of the document. The same survivor remarked, “It looks good to me…I mean, you’ve got all the boxes there.” While this evaluation may not be the most detailed, the principle of “less is more” applies when condensing a comprehensive document designed to address the needs of everyone involved. Receiving feedback like this is vital.

One survivor noted how easily he was able to understand the document, while preparing him for the future: “Oh, it was pretty much straightforward andinformative. As I mentioned, the informer was better equipped and prepared to know what I have to go through and the follow-up care.” This survivor had an upcoming appointment due to recurrence and believed that the information presented to him in the survivorship care plan would prepare him for the conversation he was going to have with his urologist. This leads researchers to believe that an enhanced quality of life is possible even when faced with recurrence.

## Discussion

This study is the first to develop and evaluate a culturally tailored SCT template specifically designed for Black men. The clinically and culturally informed SCT template that addresses the unique needs of Black prostate cancer survivors, including knowledge enhancement, cancer literacy, follow-up care, surveillance, health-related quality of life (HRQOL), health advisories, and access to culturally relevant health and support resources.

The evaluation of the SCT yielded positive results regarding a culturally tailored intervention. Survivors expressed their appreciation for the care and detail that went into crafting the SCT. This final SCT was piloted with 22 Black CaPS who acknowledged the comprehensiveness of the SCT. The feedback from survivors was incorporated into the enhancements of the CaP-SCT. Throughout the interview, survivors demonstrated remarkable openness, especially in relation to the social determinants of health (SDOH) evaluation. Their candidness not only shaped the development of each section of the enhanced SCT but also enriched numerous qualitative codes, providing deeper insights into the psychosocial and emotional well-being needs of survivors. Many expressed enthusiasm for an intervention tool specifically tailored for Black men, prompting a more thoughtful and thorough evaluation of the SCT from the survivors’ perspectives.

They endorsed the sections addressing major comorbidities, survivorship needs with guidance on provider communication, and the health advisories validated their unmet needs. They recognized that their physicians might not have the time to inquire about issues beyond prostate cancer. Having a living document could effectively help them document their concerns and provide guidance on self-care and self-management. Their approval of the survivor concerns encourages men to be more forthcoming with their emotional and mental health, something that Black men struggle to reveal [32–34].

## Study limitations

This study has some limitations. First, the sample size of this study was relatively small due to limited time and funding. Also, interview bias is one limitation of this study as we used semi-structured interviews.

## Conclusions

This study is the first to develop and evaluate a clinically and socioecologically responsive prostate cancer survivorship care toolkit (SCT) template specifically designed for a priority, prostate cancer-burdened population. The resulting SCT template addresses patient activation, cancer literacy, follow-up care, surveillance, health-related quality of life (HRQOL), health advisories, and access to culturally relevant health and support resources.

Survivors expressed favorable evaluation with high acceptance and utility of the SCT template as a credible, patient-driven tool with a clinically and community-responsive resource to improve follow-up and survivorship care, and patient documentation, activation, and communication about their survivorship concerns and needs.

## Data Availability

No datasets were generated or analysed during the current study.
